# Aerobic Exercise Attenuates Pressure Overload-Induced Cardiac Dysfunction through Promoting Skeletal Muscle Microcirculation and Increasing Muscle Mass

**DOI:** 10.1155/2021/8279369

**Published:** 2021-11-15

**Authors:** Ling-Yan Yuan, Pei-Zhao Du, Min-Min Wei, Qi Zhang, Le Lu, Xu Tian, Shao-ting Fu, Xiao-Li Zeng

**Affiliations:** ^1^Department of Kinesiology, Institute of Physical Education, Shanghai Normal University, Shanghai, China; ^2^Department of Cardiovascular Medicine, Shanghai Baoshan Hospital of Integrated Traditional Chinese and Western Medicine, Shanghai, China; ^3^Shanghai Kangjian Foreign Language Experimental Middle School, Shanghai, China; ^4^Shanghai University of Sports, Shanghai, China; ^5^Shanghai United International School, Shanghai, China

## Abstract

**Background:**

Aerobic exercise has been proven to have a positive effect on cardiac function after hypertension; however, the mechanism is not entirely clarified. Skeletal muscle mass and microcirculation are closely associated with blood pressure and cardiac function.

**Objective:**

This study was designed to investigate the effects of aerobic exercise on the skeletal muscle capillary and muscle mass, to explore the possible mechanisms involved in exercise-induced mitigation of cardiac dysfunction in pressure overload mice.

**Methods:**

In this study, 60 BALB/C mice aged 8 weeks were randomly divided into 3 groups: control (CON), TAC, and TAC plus exercise (TAE) group and utilized transverse aortic constriction (TAC) to establish hypertensive model; meanwhile, treadmill training is used for aerobic exercise. After 5 days of recovery, mice in the TAE group were subjected to 10-week aerobic exercise. Carotid pressure and cardiac function were examined before mice were executed by Millar catheter and ultrasound, respectively. Muscle mass of gastrocnemius was weighed; cross-sectional area and the number of capillaries of gastrocnemius were detected by HE and immunohistochemistry, respectively. The mRNA and protein levels of VEGF in skeletal muscle were determined by RT-PCR and western blot, respectively.

**Results:**

We found that ① 10-week aerobic exercise counteracted hypertension and attenuated cardiac dysfunction in TAC-induced hypertensive mice; ② TAC decreased muscle mass of gastrocnemius and resulted in muscle atrophy, while 10-week aerobic exercise could reserve transverse aortic constriction-induced the decline of muscle mass and muscle atrophy; and ③ TAC reduced the number of capillaries and the protein level of VEGF in gastrocnemius, whereas 10-week aerobic exercise augmented the number of capillaries, the mRNA and protein levels of VEGF in mice were subjected to TAC surgery.

**Conclusions:**

This study indicates that 10-week aerobic exercise might fulfill its blood pressure-lowering effect via improving skeletal muscle microcirculation and increasing muscle mass.

## 1. Introduction

Hypertension is a potential risk factor for cardiovascular disease [1]; long-standing elevated blood pressure induces cardiac remodeling and dysfunction, including left ventricular hypertrophy (LVH) and reduced ejection fraction (EF), ultimately leading to heart failure [2, 3]. Effective control of blood pressure can delay or even avoid the occurrence of heart failure. With an increasing number of people suffering from hypertension worldwide, searching for more effective therapeutic methods to control hypertension and then improve cardiovascular function is expected. In clinical, there are pharmacological and nonpharmacological approaches to lower blood pressure, and exercise training is often considered as the cornerstone of nonpharmacological therapy for patients with hypertension. Accumulating evidences from human and animal experiments also demonstrated that moderate intensity exercise could lower blood pressure and improve cardiac function and effectively [1, 4–6]. However, the mechanisms about the positive effects of exercise on cardiac function are not totally clarified.

Physiologically, the heart can adapt to chronic exercise in order to meet the enhanced oxygen demand of the body, a process called “remodeling”. Regular exercise can lead to a significant decrease in resting heart rate, a significant increase in maximum oxygen uptake (VO_2max_), and left ventricular mass [7]. Therefore, aerobic exercise can promote physiological cardiac hypertrophy and can contribute to preserved cardiac function. At present, many studies have confirmed that exercise can improve ventricular remodeling, which molecular mechanisms involved mainly include activation of extracellular and intracellular signal pathways, regulation of gene changes through microRNA, and so on [8]. However, most studies focus on the heart itself; considering the human body as a systematic whole, exercise on the reduction of blood pressure and the improvement of cardiac function not only can focus on the role of the heart.

Recently, it has been reported that there is signal transmission between skeletal muscle and myocardial cells. After exercise, skeletal muscle can secrete cytokines and act on the myocardium with blood circulation so as to prevent cardiac dysfunction after myocardial infarction [9]. In addition, low relative skeletal muscle mass is a major predictor of hypertension [10], and interventions that increase muscle mass will mitigate cardiovascular disease [11]. Therefore, the role of skeletal muscle should not be ignored in the study of exercise improving cardiac function.

The determinants of hypertension include effective blood volume, myocardial contractility, and peripheral vascular resistance, among which peripheral vascular resistance is closely related to skeletal muscle. Peripheral resistance is mainly affected by microcirculation, the blood circulation in capillaries between arterioles and venules. Microcirculation alterations are associated with the occurrence and development of hypertension, and multiple drugs targeting microcirculation could significantly lower blood pressure [12]. Skeletal muscle contains a dense capillary network, which transports nutrients and oxygen to meet the energy demands and removes wastes products from skeletal muscle cells. Adequate skeletal muscle capillarization is not only beneficial to reduce peripheral resistance but also can provide enough amino acids and growth factors for skeletal muscle, thus promoting protein synthesis and muscle hypertrophy [13, 14]. Individuals with hypertension often exhibit microcirculation impairment [15]. Accumulating evidence showed that exercise, no matter resistance exercise or endurance exercise, could increase skeletal muscle capillarization [16] and improve muscle hypertrophy [17, 18]. In addition, the regular contraction and relaxation of skeletal muscle also function as a pump to offset gravity, which helps blood reflux to the heart.

Vascular endothelial growth factor (VEGF) is a key regulator of angiogenesis, which plays a crucial role in the establishment of a new capillary network. The inhibition of VEGF reduced microvessel number and led to the increase of hypertension incidence during anti-cancer therapy, whereas enhancing the activity of endogenous VEGF by intramuscular local or systemic injection of small VEGF-binding peptide under hind limb ischemia and wound conditions is a common strategy to increase angiogenesis in vivo [19]. Studies have shown that in subjects with hypertension, the levels of VEGF protein and capillary density in skeletal muscle are lower than normotensive controls [20, 21], whereas exercise could normalize skeletal muscle vascular endothelial growth factor levels and lower blood pressure [20].

Transverse aortic constriction (TAC) is an accepted and valuable model to study the molecular mechanisms of heart failure due to its less invasive and time-consuming. Additionally, its lower surgical mortality, replicability, and gradual progression to cardiac failure make it a widely used model [22]. In the present study, we established the model of hypertension using TAC surgery and exerted 10-week aerobic exercise to study the effect of aerobic exercise on the blood pressure in hypertensive mice induced by TAC and explore whether the regulation of aerobic exercise on blood pressure and cardiac function was associated with skeletal muscle capillary and muscle hypertrophy?

## 2. Materials and Methods

### 2.1. Animals and Groups

Male BALB/C wild-type mice (8 weeks old; *n* = 60) were kindly provided by Tongji University (20–30 g body weight). They were housed five per cage with a 12 h light:12 h dark cycle and fed with food and water ad libitum. After acclimating to laboratory conditions for 3 days, mice were randomly divided into three groups (SPSS version 20.0; random number, 20 mice per group): control (CON), transverse aortic constriction (TAC), and transverse aortic constriction plus exercise (TAE). All the experimental procedures were performed in accordance with the Ethics Committee of Shanghai Normal University (Ethics Committee registration number: 2019shnu116).

### 2.2. Establishment of Pressure Overload Mice Model

The pressure overload mice model was established by transverse aortic constriction (TAC) surgery or sham operation according to previous studies [23, 24]. In brief, surgery in mice was performed under anesthesia by isoflurane; when the mice manifested as having no corneal reflex, weakened limbs, and paralysis, and so on, they were placed in a supine position and fixed onto a wooden board. Immediately afterwards, endotracheal intubation was performed, and the animals were maintained under mechanical ventilation, with a tidal volume of 2.5–3 mL/min (ambient air) and a respiratory rate of 90–110 breaths per minute. Then thoracotomy was conducted, with an incision between the second and third left intercostal space, and the thymus was retracted to expose the transverse aorta, which is located between the innominate and left carotid arteries. After that, a 27-gauge needle was positioned perpendicular to the vessel, which served as a template to determine the degree of constriction after a double knot was given, and a cardiovascular suture wire (7.0 TI-CRON) was used to perform the constriction. The needle was removed, and the suture was performed. Mice in the CON group underwent the same procedures as above but without the constriction of the transverse aorta.

### 2.3. Exercise Protocol

After 5 days of TAC operation recovery and another 5 days of adaptation to a treadmill, mice in the TAE group received 10-week treadmill exercise with increasing load, 5 days per week. In the first week, mice were trained at 12 m/min and 30 min/day, and the speed and duration were gradually increased up to 15 m/min and 60 min/day at the beginning of the sixth week and then maintained for 5 weeks, which was performed in on a motor-driven treadmill (size of 565*∗*630*∗*310 mm and single runway width 55 mm, Hangzhou, China); the treadmill always keeps 0% grade in this exercise (Supplementary Figure S1). The mouse sat in the treadmill chamber for 10 min before running commenced, during which time grooming and exploration usually occurred. Treadmill speed was increased in 2 min intervals by 5 m/min until the assigned speed was reached. Moreover, in order to avoid the impact of circadian rhythm on cardiovascular response, all training is from 10 a.m. to 11 a.m.

### 2.4. Determination of Carotid Pressure

To verify whether the pressure overload model was established successfully, the carotid pressure of mice before tissue collection was examined. Briefly, mice were anesthetized intraperitoneally using 1.5% pentobarbital sodium solution (0.1 ml/20 g weight). Then an incision was cut along the middle line of the neck to expose the trachea, and the right common carotid artery was detached. The concentric end of the right common carotid artery was clamped; a “V” shaped incision was cut in the right common carotid artery; and Millar catheter was thrust in the left ventricle from the “V” shaped incision in the right common carotid artery; meanwhile, blood pressure waveform was examined. When the blood pressure stabilized, the Millar catheter was ligated and fixed using suture wire threaded in advance. Aortic systolic pressure was examined and recorded when blood pressure was maintained for 5 min.

### 2.5. Cardiac Ultrasound

Cardiac function was determined using VEVO 770 Diagnostic Ultrasound System (VisualSonics, Toronto, Canada). Mice were anesthetized using isoflurane following a fast, and their hair was removed using depilatory paste. Then mice were fixed onto the heating plate in a supine position to maintain body temperature, respiratory assistance system, and anesthesia system. Medical ultrasonic couplant was scrawled on precordium; parasternal long- and short-axis on thoracic anterior were collected; and electrocardiographic and M-mode images were recorded by 707B 30 MHz probe. And left ventricular internal dimension systole (LVIDs), left ventricular internal dimension diastole (LVIDd), and left ventricular ejection fraction (EF, %) were measured.

### 2.6. Muscle Histology

The muscles were dissected free from surrounding connective tissue, weighed, and embedded in paraffin wax. All samples were sectioned 5 *μ*m thick using a rotary microtome (Leica, Germany); muscle tissue sections were stained with hematoxylin-eosin (HE) to calculate the cross-sectional area (CSA) of muscle fiber. Five view fields of each section were chosen randomly, and the morphology of sections was analyzed by the light microscope at 40× lens objective. The total area of myocytes and the number of myonuclei were also analyzed by Leica Qwin Plus software, and the cross-sectional area of myocyte was calculated.

For immunochemistry, paraffin sections were deparaffinized and rehydrated. Rehydrated sections underwent an antigen retrieval process, were blocked with immunol staining blocking buffer (Beyotime Biotechnology, Shanghai, China) at RT for 30 min, and then incubated with primary antibody against CD31 overnight at 4℃ at a dilution of 1:50 (BOSTER Biological Technology, Wuhan, China). After washing three times with PBS, 12 min/time, sections were then incubated with a biotin-labeled secondary antibody at 37°C for 30 min. After washing three times again, sections were incubated with streptavidin-biotin complex (SABC) containing HRP at 37°C for 30 min; then DAB solution was dropped onto sections after washing four times, 15 min/time. Then sections were restained with hematoxylin and covered with neutral resins. The sections were morphologically analyzed by the light microscope at 40× lens objective; five view fields of each section were chosen randomly; and the number of vascular was calculated.

### 2.7. Real-Time Quantitative PCR

Total RNA was extracted using Trizol reagent and transcribed into cDNA using a commercially available kit following the manufacturer's instructions. Then target genes were amplified using SYBR Green Realtime Master Mix. Primers for amplification genes of VEGF (sense: 5′-GAAGAAAGTGGTGCCATGGATAG-3′, antisense: 5′-CCCATGAGTTCCATGCTCAGA-3′) and GAPDH (sense: 5′-ACCACAGTCCATGCCATCA C-3′, antisense: 5′- TCCAC CACCCTGTTGCTGTA-3′) were synthesized by Sangon Biotech Co. Ltd. The amplification conditions for these genes were as follows: 60 s denaturation at 95°C followed by 40 cycles of 15 s denaturation at 95°C, 60 s annealing, and elongation at 60°C. The mRNA values of VEGF of samples were corrected by that of the internal control of GAPDH and shown as the ratios of target genes to GAPDH.

### 2.8. Western Blotting

About 30 mg gastrocnemius was cut into small pieces and homogenized after adding 300 *μ*l radioimmunoprecipitation assay (RIPA) buffer containing phenylmethanesulfonyl fluoride (PMSF) to extract total protein. Lysates were then centrifuged at 12,000 rpm for 20 min at 4°C followed by standing on ice for 30 min. The supernatants were collected, and protein concentration was determined by a BCA protein assay kit (Bio-Rad Laboratories, Hercules, USA). A total of 50 *μ*g protein was electrophoresed on 10% SDS-PAGE gels, then transferred onto polyvinylidene fluoride (PVDF) membrane and blocked with 5% nonfat milk at room temperate for 1 h. Then the PVDF membranes were incubated with primary antibodies against VEGF (Millipore, USA) and GAPDH (Cell Signaling Technology, USA) at 4°C overnight. Then the bands were washed three times with TBST, 15 min/time, and incubated with horseradish peroxidase (HRP) conjugated secondary antibody at room temperate for 2 h. Subsequently, the membranes were washed four times, 5 min/time, before visualization by automatic chemiluminescence apparatus, and the density of the bands was analyzed by Image-Pro Plus software.

### 2.9. Statistical Analysis

All data were presented as mean standard deviation (mean ± SD), and statistical analysis of the data was performed using SPSS 17.0 software package; multiple groups were compared by one-way ANOVA followed by LSD procedure; *p* < 0.05 was considered as a significant difference; and *p* < 0.01 was considered as a very significant difference (effect size were calculated and listed in Supplementary Table S2).

## 3. Results

### 3.1. Aerobic Exercise Attenuated TAC-Induced Hypertension

**Figure 1:**
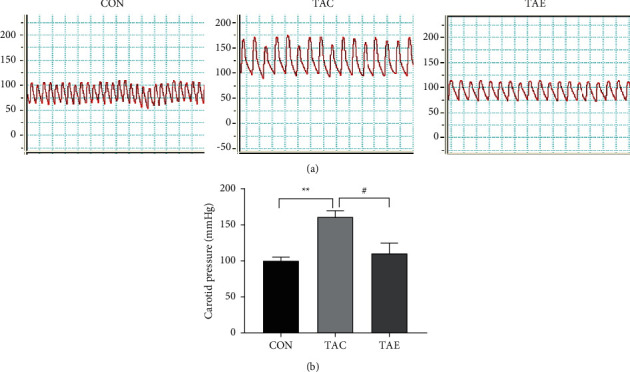
Exercise attenuated TAC-induced hypertension. The carotid pressure was determined after exercise was finished by Millar catheter. Compared with the CON group, TAC surgery significantly increased the carotid blood pressure, indicating the successful establishment of hypertension; compared with the TAC group, the carotid blood pressure of the TAE group was lowered, which suggested that aerobic exercise was an effective method to prevent elevated blood pressure. CON: control, TAC: transverse aortic constriction, and TAE: transverse aortic constriction plus exercised. *n* = 6–7 per group.  ^*∗∗*^*p* < 0.01 vs. CON; ^#^*p* < 0.05 vs. TAC.

To verify the efficiency of hypertension establishment and the effects of exercise on the blood pressure in TAC-induced hypertensive mice, we detected the carotid blood pressure by Millar catheter. As shown in Figure 1, TAC surgery led to the increase of blood pressure significantly; 10-week aerobic exercise prevented the elevated blood pressure, which further confirmed that aerobic exercise could be an effective intervention to lower blood pressure.

### 3.2. Aerobic Exercise Mitigated Cardiac Structure and Dysfunction of TAC-Induced Hypertensive Mice

**Figure 2:**
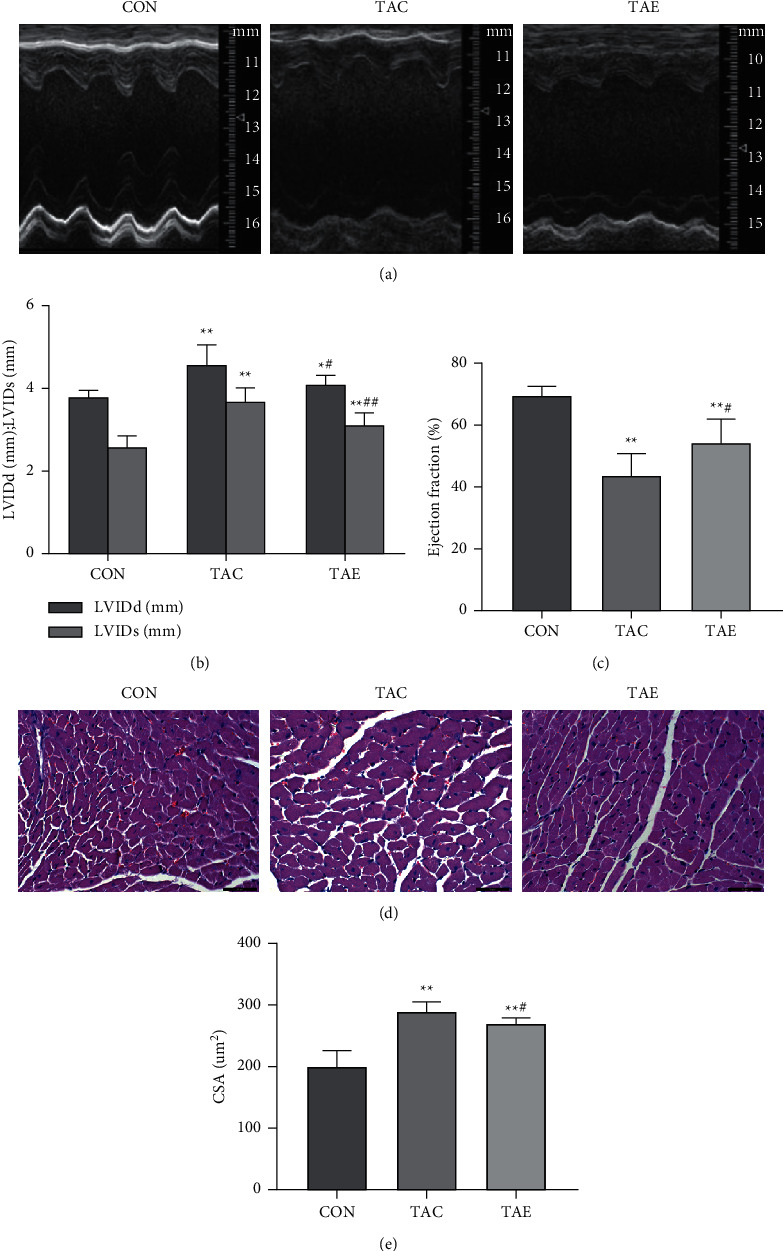
Exercise mitigated cardiac structure and dysfunction in TAC-induced hypertensive mice. Cardiac LVIDs, LVIDd, and ejection fraction were detected by ultrasound; the respective pictures of cardiac ultrasound in CON, TAC, and TAE were shown (a), and the results of LVIDs, LVIDd, and ejection fraction were calculated (b, c), heart muscle cross-sectional area was determined by HE staining, five views of each section were chosen, and pictures were captured by light microscope at 400× magnification (d). Total area and the number of cell nuclear were examined by Leica Qwin Plus software, and the muscle cross-sectional area was calculated (e). CON: control, TAC: transverse aortic constriction, and TAE: transverse aortic constriction plus exercised. *n* = 6 per group. ^*∗*^*p* < 0.05 vs. CON;  ^*∗∗*^*p* < 0.01 vs. CON; ^#^*p* < 0.05 vs. TAC.

To determine the effects of long-term hypertension and exercise on cardiac structure and function, we measured LVIDs, LVIDd, and ejection by ultrasound (Supplementary Table S1) and heart muscle cross-sectional area by HE staining. As shown in Figure 2, we found that TAC-induced hypertension increased LVIDs, LVIDd (Figures 2(a) and 2(b)), and heart muscle cross-sectional area (Figures 2(d) and 2(e)), and reduced left ventricular ejection fraction (Figure 2(c)) and 10-week aerobic exercise decreased LVIDs, LVIDd, and heart muscle cross-section, and increased ejection fraction, although LVIDs, LVIDd, and heart muscle cross section still higher than that of mice in CON group, and ejection fraction still lower than that of mice in CON group (Figure 2).

### 3.3. Aerobic Exercise Augmented the Number of Skeletal Muscle Capillaries in TAC-Induced Hypertensive Mice

**Figure 3:**
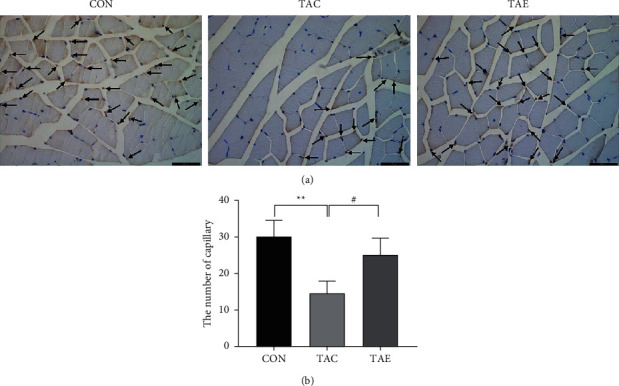
Aerobic exercise augmented the number of skeletal muscle capillaries in TAC-induced hypertensive mice. Skeletal muscle capillaries were determined by immunostaining with anti-CD31 antibody, platelet endothelial cell adhesion molecule-1. The positive spots that the arrows indicated represented capillary (a). Five views of each section were chosen, and the number of capillaries were counted under the light microscope at 400× magnification and analyzed by independent *t*-test (b). CON: control, TAC: transverse aortic constriction, and TAE: transverse aortic constriction plus exercised. *n* = 6 per group.  ^*∗∗*^*p* < 0.01 vs. CON; ^#^*p* < 0.05 vs. TAC.

Skeletal muscle microvascular rarefaction is associated with hypertension. In the present study, we examined the effects of exercise on the number of skeletal muscle microvascular to study the mechanisms with regard to exercise-induced blood-pressure-lowering effect. As shown in Figure 3, TAC significantly reduced the number of skeletal muscle capillaries, while aerobic exercise remarkably increased the number of skeletal muscle capillaries in TAC-induced hypertensive mice, indicating that exercise-induced blood-pressure-lowering effects might be associated with the improvement of skeletal muscle microcirculation.

### 3.4. Aerobic Exercise Increased the mRNA and Protein Levels of VEGF in Skeletal Muscle of TAC-Induced Hypertensive Mice

**Figure 4:**
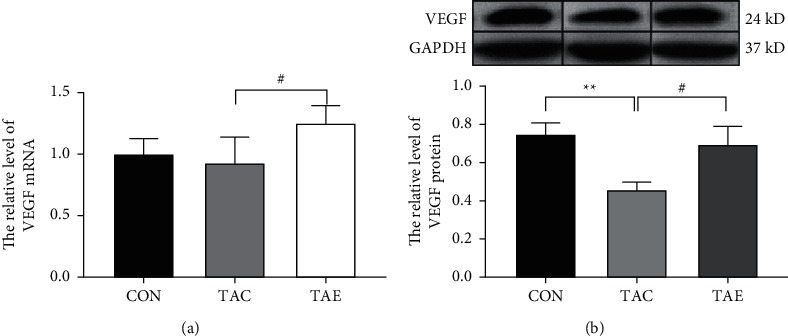
Aerobic exercise increased the mRNA and protein levels of VEGF in skeletal muscle of TAC-induced hypertensive mice. The relative mRNA (a) and protein (b) levels of VEGF in skeletal muscle were examined by RT-PCR and western blot, respectively. CON: control, TAC: transverse aortic constriction, and TAE: transverse aortic constriction plus exercised. *n* = 6 per group.  ^*∗∗*^*p* < 0.01 vs. CON; ^#^*p* < 0.05 vs. TAC.

To verify whether exercise increased the number of capillaries in skeletal muscle was associated with VEGF, we detected the mRNA and protein levels of VEGF. As shown in Figure 4, compared with the CON group, the protein level of VEGF in skeletal muscle was significantly reduced in the TAC group (Figure 4(b)). Compared with the TAC group, the mRNA and protein levels of VEGF were remarkably increased in TAE group (Figures 4(a) and 4(b)).

### 3.5. Aerobic Exercise Totally Reversed Relative Skeletal Muscle Mass Loss and Alleviated Muscle Atrophy Induced by TAC

**Figure 5:**
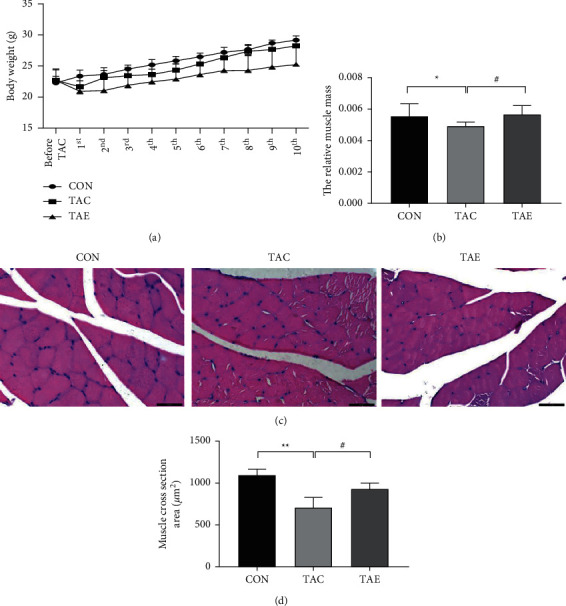
Aerobic exercise totally reversed relative skeletal muscle mass loss and alleviated muscle atrophy induced by TAC. (a) The body weight was weighed at different time points after TAC surgery. (b) The value of relative skeletal muscle mass was calculated by the formula: = the mass of gastrocnemius divided by body weight. (c) Skeletal muscle morphology was determined by HE staining; five views of each section were chosen; and pictures were captured by light microscope at ×400 magnification. (d) Total area and the number of cell nuclear were examined by Leica Qwin Plus software, and the muscle cross-sectional area was calculated. CON: control, TAC: transverse aortic constriction, and TAE: transverse aortic constriction plus exercised. *n* = 6 per group. ^*∗*^*p* < 0.05 and  ^*∗∗*^*p* < 0.01 vs. CON; ^#^*p* < 0.05 vs. TAC.

Capillaries have the important function of delivering oxygen, nutrients, and hormones to tissues. Adequate skeletal muscle capillarization can enhance muscle protein synthesis by ensuring the transport of amino acids and growth factors to muscle fibers, thus promoting muscle hypertrophy and the increase of muscle mass. In the current study, we determined the effects of TAC and TAE on relative muscle mass and cross-sectional area. As shown in Figure 5, despite TAC and exercise had no effects on body weight (Figure 5(a)), TAC led to the decline of relative skeletal muscle mass (Figure 5(b)) and cross-sectional area (Figure 5(d)), whereas 10-week aerobic exercise totally reversed muscle loss (Figure 5(b)) and promoted muscle hypertrophy (Figure 5(d)) induced by TAC.

## 4. Discussion

Aerobic exercise has a positive preventive and therapeutic effect on chronic diseases. The grasp of the volume and intensity of exercise has become the restrictive factor to obtain reasonable experimental results. Treadmill training is a commonly used exercise model in sports experiments. Treadmill running imposes potent metabolic stress on the animal, requiring significant increases in O_2_ delivery to the contracting skeletal muscles in intensity- and time-dependent manners. Treadmill training of mice increases VO_2max_, maximal running speeds, endurance capacity, heart and ventricular masses, skeletal muscle mass, and capillarization. The research of Canadian scholar Fernando and his colleagues provides a reliable and simple method for judging the submaximal aerobic exercise and corresponding exercise intensity of mice. Their results showed that for mice weighing 19–28 g, the relationship between speed (0% grade, 10–20 m/min) and VO_2max_ could be expressed as *y* = 62.911 + 1.1276×. The VO_2max_ corresponding to the running speed of 12–15 m/min is between 75% and 80%. Because mice use more than 50% of their maximum capacity to transport oxygen at rest, their tranquility rate is much higher than that of large animals. For mice, the exercise protocol used in this experiment represents moderate intensity exercise [25].

Consistent with previous studies, we demonstrated that aerobic exercise lasting for 10 weeks effectively prevented the elevated blood pressure in TAC-induced hypertensive mice; furthermore, exercise attenuated cardiac dysfunction and mitigated TAC-induced pathological ventricular remodeling, which suggested that aerobic exercise was an effective intervention for hypertension and prevented the development of heart failure. Our results indicate that the beneficial effect may be related to the increase of capillaries and skeletal muscle hypertrophy.

As mentioned above, peripheral vascular resistance is one of the important factors in the formation of blood pressure, and reducing peripheral vascular resistance can effectively reduce blood pressure. Exercise training affects blood pressure mostly by the change of vascular function and structure eventually leading to decreased peripheral resistance [26], and the role of the capillary network cannot be ignored. Physiologically, microcirculation composed of the capillary network plays an important role in determining peripheral vascular resistance. Sufficient capillaries ensure the flow efficiency of arteriovenous blood, prevent the increase of peripheral vascular resistance, and maintain stable blood pressure. Other studies have found that microvascular rarefaction is considered to be highly associated with the development of hypertension [27]. In several tissues, capillary density was shown to be inversely correlated with blood pressure [28]. Furthermore, capillary rarefaction in skeletal muscle is associated with the elevation of arterial pressure in hypertensive individuals [29], and individuals with hypertension are often accompanied by skeletal muscle capillary rarefaction [15].

Skeletal muscle capillarization plays a key role in oxygen and nutrient delivery to muscle. Skeletal muscle capillarization rarefaction is one of the factors that may contribute to muscle atrophy during aging [30]; skeletal muscle mass is positively correlated with capillarization. Low relative skeletal muscle mass predicts the incidence of hypertension [10, 31], whereas high muscle mass protects against obesity-induced hypertension [11]. Regular exercise, as an effective approach to promote skeletal muscle hypertrophy [17, 18], was shown to reduce blood pressure and improve capillary density in an obese rat with metabolic syndrome [32]. However, the role of long-term aerobic exercise-induced muscle hypertrophy in lowering blood pressure has not been reported. In the present study, we found that 10-week aerobic exercise attenuated TAC-induced capillary rarefaction in skeletal muscle and simultaneously promoted muscle hypertrophy, one of the best factors to study in this scenario is VEGF.

It is well known that VEGF is an important regulator of angiogenesis, as evidenced by a severely impaired vascular network in skeletal muscle of VEGF knockout mice [20, 33]. Under a sedentary state, hypertensive individuals have a lower level of VEGF protein and capillary density in skeletal muscle compared to controls [20], whereas exercise training normalizes their level of VEGF protein [20] and capillary density [34–36]. Consistently, we found that TAC surgery significantly reduced the level of VEGF protein and the number of capillaries in skeletal muscle, whereas regular aerobic exercise reversed the above process. All data suggested that aerobic exercise could attenuate TAC-induced microvascular rarefaction in skeletal muscle, which may be achieved by increasing the level of VEGF.

Vascular effects of exercise are based on improved endothelium-mediated flow-induced vasodilatation in conduit arteries and larger resistance arteries, higher myogenic control, and increased metabolic vasodilatation in small resistance arteries. Vascular regeneration by mobilization of endothelial progenitor cells is augmented by exercise [37], which is consistent with our results. We did not discuss the role of metabolic factors such as eNOS and ROS because we focus on the number of blood vessels. The number of microcirculation and the regulation of vascular endothelial function are two relatively independent aspects.

**Figure 6:**
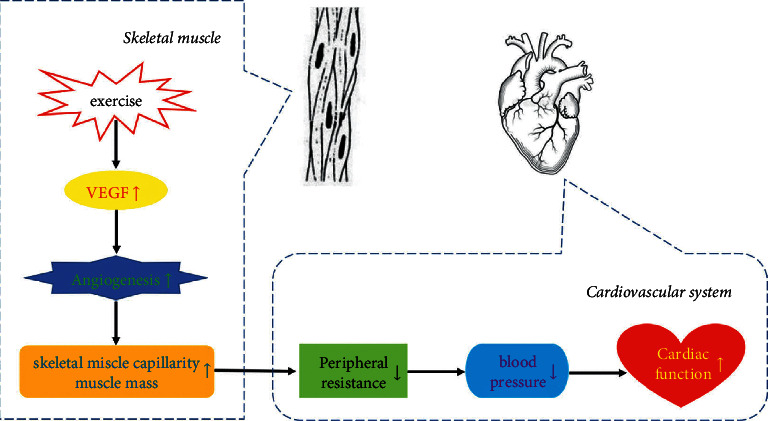
Proposed mechanism underlying the effects of aerobic exercise attenuates pressure overload-induced cardiac dysfunction.

For decades, we have known that exercise training exerts beneficial effects on the human body. In order to better incorporate physical exercise into the treatment plan of patients with cardiovascular disease, the mechanism of exercise prescription to treat cardiovascular diseases urgently needs to be further clarified. Our research shows that exercise promotes the expression of VEGF to increase microcirculation by skeletal muscle capillarity and muscle mass, thereby reducing peripheral resistance and attenuating blood pressure, which prevents cardiac dysfunction eventually (Figure 6). To our knowledge, this is the first report on aerobic exercise-induced muscle hypertrophy in lowering blood pressure in the TAC-induced hypertensive mice model, which provided a new approach to study the mechanisms underlying the lowering blood pressure effect of long-term aerobic exercise.

Our experiment is based on a continuous endurance training model, so it is not known whether our results are applicable to other training modalities. What's more, the intrinsic molecular mechanism that exercise promotes VEGF expression needs to be further studied, and the neuroendocrine mechanism may be a direction.

## 5. Conclusions

In summary, we found that TAC led to capillary rarefaction in skeletal muscle and muscle atrophy and that 10-week aerobic exercise increased capillary density in skeletal muscle and prevented muscle atrophy in TAC-induced hypertensive mice. All our results suggested that the lowering blood pressure effect of long-term aerobic exercise on TAC-induced hypertensive mice might be associated with the mitigation of capillary rarefaction in skeletal muscle and muscle hypertrophy, which provided a new explanation for lowering blood pressure effect of aerobic exercise in hypertensive mice model.

## Data Availability

The data used to support the findings of this study are available from the corresponding author upon request.

## Abbreviations

CON: Control
EF: Ejection fraction
eNOS: Endothelial nitric oxide synthase
HE: Hematoxylin-eosin
HRP: Horseradish peroxidase
LVH: Left ventricular hypertrophy
LVIDd: Left ventricular internal dimension diastole
LVIDs: Left ventricular internal dimension systole
PMSF: Phenylmethanesulfonyl fluoride
PVDF: Polyvinylidene fluoride
RIPA: Radioimmunoprecipitation assay
ROS: Reactive oxygen species
RT-PCR: Reverse transcription-polymerase chain reaction
TAC: Transverse aortic constriction
TAE: Transverse aortic constriction plus exercise
VEGF: Vascular endothelial growth factor
VO_2max_: Maximum oxygen uptake.
