# Supplementary material for: Aerobic Exercise Attenuates Pressure Overload-Induced Cardiac Dysfunction through Promoting Skeletal Muscle Microcirculation and Increasing Muscle Mass
Source: Evid Based Complement Alternat Med. 2021 Nov 15;2021:8279369. doi: 10.1155/2021/8279369 (PMC8608514; doi:10.1155/2021/8279369)
Supplement: Supplementary Materials — Supplementary data associated with this article can be found in the supplemental files. [file 8279369.f1.docx]

**Figure.S1 The flowchart and exercise protocol of the experiment.**


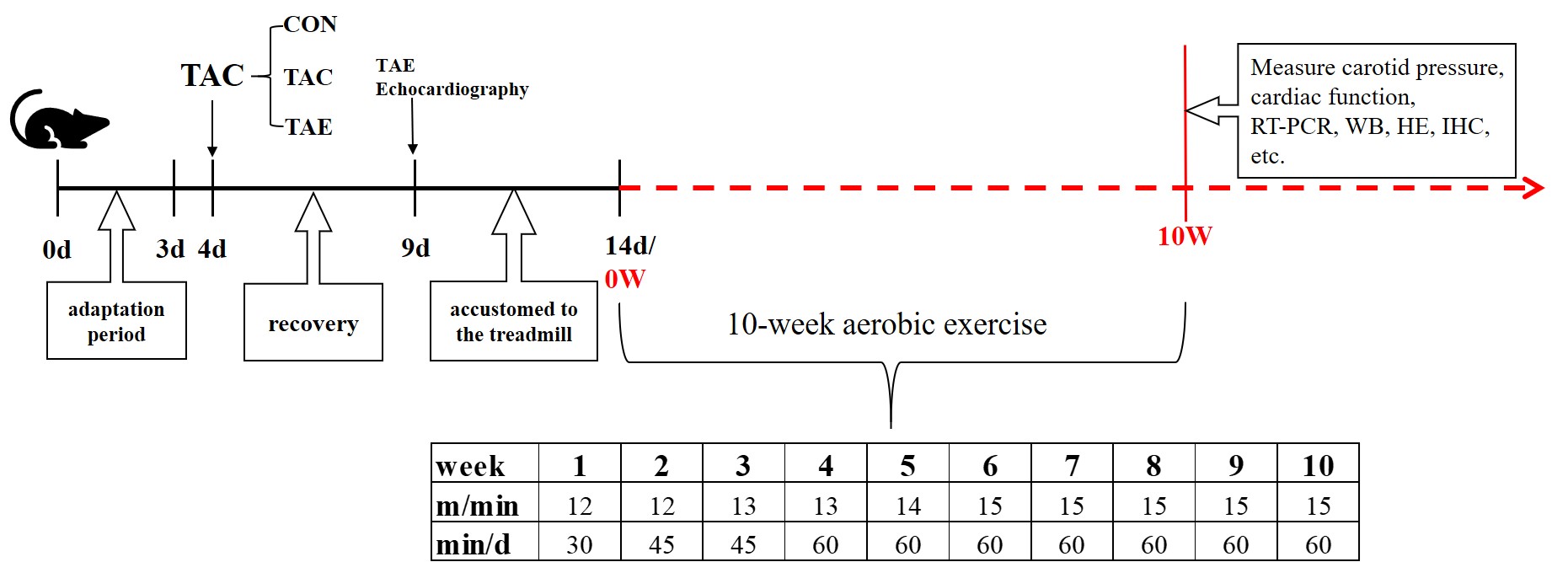


**Table.S1 Measurement results of ultrasonic indexes in different groups of mice.**

*p<0.01 vs. CON; ** p<0.01 vs. CON;^p<0.01 vs. TAC-baseline; ^^ p<0.01 vs. TAC-baseline; # p<0.05 vs. TAC; ## p<0.01 vs. TAC; + p<0.05 vs. TAE-baseline; ++ p<0.01 vs. TAE-baseline.

|  | **HR(bpm)** | **LVEF(%)** | **LVIDd(mm)** | **LVIDs(mm)** |
| --- | --- | --- | --- | --- |
| **CON** | **470±42.4** | **69.5±3.1** | **3.78±0.18** | **2.57±0.28** |
| **TAC-baseline** | **468±41.0** | **67.5±3.3** | **3.75±0.17** | **2.55±0.32** |
| **TAC** | **500±47.5** | **43.6±7.2**^^++** | **4.56±0.50**^^++** | **3.68±0.35**^^++** |
| **TAE-baseline** | **473±40.5** | **68.6±3.6** | **3.79±0.20** | **2.50±0.34** |
| **TAE** | **483±47.6** | **54.3±8.3**^^++#** | **4.08±0.25*^+#** | **3.10±0.31**^^++##** |

**Table.S2 Effect size of results in each section.**

| **Results** | **η²** |
| --- | --- |
| carotid pressure | 0.963 |
| LVIDd | 0.812 |
| LVIDs | 0.994 |
| EF | 0.973 |
| CSA | 0.958 |
| the number of capillary | 0.953 |
| VEGF mRNA | 0.939 |
| VEGF protein | 0.978 |
| the relative muscle mass | 0.989 |
| muscle cross section | 0.988 |
